# Pathological Society of Philadelphia

**Published:** 1882-10

**Authors:** H. F. Formad


### PROCEEDINGS OF SOCIETIES.

### PATHOLOGICAL SOCIETY OF PHILADELPHIA, MAY 25, 1882.

### CANCER OF MAMMARY GLAND OF DOG, WITH METASTASIS TO LIVER AND LYMPHATIC GLANDS. EXHIBITED BY DR. H. F. FORMAD.

An old female rat-dog about two years ago showed a circumscribed induration in one of the mammary glands of the size of a walnut. This subsequently proved to be a permanent tumor, increasing very slowly in size, and causing the animal to suffer pain at times intense. One year ago, when I saw it first, the tumor was of the size of a hen's egg, and painful on pressure. There was also involvement of the axillary glands, which were large and nodulated. I did not see the dog again until a year later, when it died and was kindly sent to me by Dr. Keys.

*Antopsy.*—The animal was greatly emaciated. The mammary tumor had reached the size of a large orange, was encapsulated, round and flat in shape, hard, skin movable over tumor. All over the abdomen and chest there were seen about twenty secondary tumor-nodes, varying in size from a pea to a hazel-nut. The axillary glands were nodular and greatly enlarged through infiltration by the new growths. Several secondary nodes of the growths were found in the liver. There was also a remarkable enlargement of the thyroid gland, on section proving an eneurismal goitre. Microscopic examination of themammary tumor and of the secondary deposits showed scirrhous cancer.

Dr. S. W. Gross remarked that if this growth was truly carcinomatous it certainly did not resemble the same neoplasm in man, as it was clearly circumscribed by a distinct capsule.

Dr. Tyson was a little doubtful whether it could be really carcinoma, even with the microscopic appearances described, since microscopically the growth differed entirely from human carcinoma, as it did not infiltrate the surrounding tissues.—*Philadelphia Medical Times.*

